# Ambient weathering of magnesium oxide for CO_2_ removal from air

**DOI:** 10.1038/s41467-020-16510-3

**Published:** 2020-07-03

**Authors:** Noah McQueen, Peter Kelemen, Greg Dipple, Phil Renforth, Jennifer Wilcox

**Affiliations:** 1 Department of Chemical Engineering, Clean Energy Conversions Lab, Worcester Polytechnic Institute, 100 Institute Road, Worcester, MA 01609 USA; 2 Department of Earth & Environmental Sciences, Lamont-Doherty Earth Observatory, Columbia University, 91 Rte 9W, Palisades, NY 10964 USA; 3 Department of Earth, Ocean, and Atmospheric Sciences, Bradshaw Research Initiative for Minerals and Mining, University of British Columbia, 2020–2207 Main Mall, Vancouver, BC V6T 1Z4 Canada; 4 Research Centre for Carbon Solutions, School of Engineering and Physical Sciences, Heriot-Watt University, Edinburgh, EH14 4AS UK

**Keywords:** Climate-change mitigation, Carbon capture and storage, Engineering

## Abstract

To avoid dangerous climate change, new technologies must remove billions of tonnes of CO_2_ from the atmosphere every year by mid-century. Here we detail a land-based enhanced weathering cycle utilizing magnesite (MgCO_3_) feedstock to repeatedly capture CO_2_ from the atmosphere. In this process, MgCO_3_ is calcined, producing caustic magnesia (MgO) and high-purity CO_2_. This MgO is spread over land to carbonate for a year by reacting with atmospheric CO_2_. The carbonate minerals are then recollected and re-calcined. The reproduced MgO is spread over land to carbonate again. We show this process could cost approximately $46–159 tCO_2_^−1^ net removed from the atmosphere, considering grid and solar electricity without post-processing costs. This technology may achieve lower costs than projections for more extensively engineered Direct Air Capture methods. It has the scalable potential to remove at least 2–3 GtCO_2_ year^−1^, and may make a meaningful contribution to mitigating climate change.

## Abstract (web summary)

To remove CO_2_ from the atmosphere every year by mid-century will need new technologies. Here the authors proposed the use of magnesia (MgO) in ambient looping processes to remove CO_2_ from the air and they found that the proposed approach will cost $46–195 tCO_2_^−1^ net removed from the atmosphere considering both grid and solar electricity resources without including post-processing costs.

## Introduction

The atmospheric concentration of CO_2_ has reached 410 parts per million by volume (ppm), an increase of almost 20 ppm in the last 10 years^1,2^. As current emission levels exceed 35 GtCO_2_ year^−1^^3,4^, a diverse portfolio of CO_2_ mitigation technologies must be developed and strategically deployed to avoid more than a 2 °C increase in Earth’s temperature^5^. Due to global reliance on fossil fuels, and because it is unlikely our global economy will completely eliminate all emissions, this portfolio will include technologies that remove CO_2_ from the atmosphere (negative emission technologies, NETs)^6–8^. Some NETs propose accelerating natural processes such as CO_2_ uptake in oceans and terrestrial carbon sinks (soils, forests, minerals), or bioenergy with carbon capture and storage. The work described here is germane to NETs that use sorbents to scrub greenhouse gases from the atmosphere, also known as direct air capture (DAC) with storage^7^. Prior to deployment of these technologies, it is important to understand their potential economic, social, political, and environmental impact. This study presents an overview of, and technoeconomic analysis for, a process that pairs enhanced weathering with calcination for the repeated use of mineral feedstocks to remove CO_2_ from the atmosphere.

Enhanced weathering, emulating and accelerating natural weathering processes, was first proposed by Walter Seifritz in 1990^9^. In natural weathering, silicate minerals are transformed into alkalinity-containing carbonate minerals on geologic timescales^10–12^. The generalized natural weathering reaction is described below in Eq. (1)^13^:

1 $${\mathrm{MeO}}\left( {{\mathrm{SiO}}_2} \right) + {\mathrm{CO}}_2 \to {\mathrm{MeCO}}_3 + \left( {{\mathrm{SiO}}_2} \right) + {\mathrm{Energy}},$$

where Me represents a divalent metal cation (typically magnesium (Mg^2+^) and calcium (Ca^2+^)), where suitable feedstocks include rocks (containing olivine and serpentine) as well as industrial byproducts, such as mine tailings and steel slag^14^. Since natural weathering occurs on geological timescales, researchers have explored methods to accelerate CO_2_ mineralization^15–19^. Conversely, in calcination, solid carbonate minerals are heated to decompose into metal oxides and CO_2_. The generalized calcination reaction is shown below in Eq. (2):

2 $${\mathrm{MeCO}}_3 + {\mathrm{Energy}} \to {\mathrm{MeO}} + {\mathrm{CO}}_2.$$

By pairing enhanced weathering with calcination, mineral feedstocks can be repeatedly used to capture and evolve CO_2_.

Systems for CO_2_ removal using carbonation reactions have been previously investigated. In 2011, the American Physical Society (APS) evaluated a system where CO_2_ is absorbed by sodium hydroxide (NaOH) and subsequently reacted with calcium hydroxide (Ca(OH)_2_) to produce solid calcium carbonate (CaCO_3_) ^2^. The CaCO_3_ is calcined in an oxy-fired calciner to release CO_2_. Keith et al.^20^ propose a continuous looping process consisting of an aqueous potassium hydroxide (KOH) sorbent coupled with a calcium caustic recovery loop. The KOH sorbent reacts with CO_2_ in air to produce aqueous potassium carbonate (K_2_CO_3_). K_2_CO_3_ then reacts with Ca(OH)_2_, produced by calcining solid CaCO_3_, to reproduce KOH and CaCO_3_ ^20^. These types of aqueous looping systems have been primarily evaluated using calcium-based sorbents^21–23^. Calcium looping systems have also been demonstrated for flue gas capture, where the CO_2_ concentration typically ranges from 4% (natural gas combined cycle) to 10–15% (pulverized coal combustion). Manovic and Anthony^24^ explored using CaO/CuO-based solid sorbents for capture of CO_2_ from concentrated streams. Additionally, Kheshgi^25^, Renforth et al.^26^, Renforth and Kruger^27^ propose an ocean liming process that deposits lime (CaO produced by calcining carbonate minerals) into the ocean to react with carbonic acid currently in the ocean. This process increases oceanic alkalinity and leads to storage of carbon in the ocean as bicarbonate ions, reducing atmospheric CO_2_ concentration. Alternative systems consider reactions between minerals and concentrated CO_2_ in high temperature/pressure reactors or through multistep extraction^28–30^.

Researchers have also investigated using magnesia (MgO) in looping processes. The calcination temperature of magnesite (MgCO_3_) is lower than CaCO_3_, with a 66% lower enthalpy of decarbonization, potentially leading to a lower energy cost. Magnesium is also attractive as there are large deposits of magnesium silicate minerals throughout the world^10,31,32^. Song et al.^33^ synthesized MgO from magnesite by varying calcination temperatures from 400 to 600 °C when calcining for 2 h. MgO synthesized at higher calcination temperatures had a lower surface area, larger pore size, and a decrease in overall CO_2_ uptake capacity. Researchers have also investigated forming MgO with properties optimized for CO_2_ capture at ambient conditions, achieving higher specific surface areas (330.5 m^2^ g^−1^) than commercially available MgO and increased CO_2_ uptake capacity (6.18 wt% at 25 °C)^34^. Additional work focused on CO_2_ uptake by mesoporous MgO promoted by sodium salts, such as NaNO_3_ and Na_2_CO_3_^35^, as well as KNO_3_^36^ demonstrating capture capacities of up to 19.8 mmol g^−1^ (or 80% of the theoretical capacity)^37,38^.

Previous investigations of Ca- and Mg-looping focused on rapid carbon mineralization, for example in fluidized bed reactors^39,40^. The costs of building and operating such reactors contribute significantly to integrated looping cost. In this analysis, to reduce costs and ensure simple scalability, we do not include an engineered sorption/desorption-based process. Instead, we focus on land-based, enhanced weathering that could remove significant quantities of CO_2_ from air at a relatively low cost, with an area requirement comparable to other potential CO_2_ removal methods. Specifically, we consider using magnesite (MgCO_3_) as the source of MgO in a looping system that removes CO_2_ from air via enhanced weathering. We show that this process could cost approximately $46–$159 tCO_2_^−1^ net removed from air, considering both grid and solar electricity resources without including postprocessing costs. This technology may achieve lower costs than optimistic projections for other more highly engineered DAC methods and may be used to remove significant amounts of CO_2_ from air.

## Results

### Process concept and overview

In this process, MgCO_3_ is calcined to produce caustic MgO and high-purity CO_2_. The MgO is spread over land to react with atmospheric CO_2_ to form magnesite (MgCO_3_) and other Mg-carbonate minerals over the course of a year. After renewed formation of Mg-carbonate minerals, the weathered material is collected and calcined again, producing a nearly pure stream of CO_2_ together with an amorphous, solid MgO residue (caustic magnesia, i.e. caustic MgO). The resulting MgO can be exposed to weathering again, and so on.

The produced MgO is assumed to have the same reactivity as mineral brucite (Mg(OH)_2_). The rate of formation of magnesium carbonate via reaction of aqueous brucite is on the order of 3 × 10^−8^ moles m^−2^ s^−1^ when mineral dissolution kinetics are rate limiting^7,41,42^. Thus, for example, grains of brucite with a diameter of 10–100 μm (1.7 × 10^−10^–1.7 × 10^−7^ moles, 1.25 × 10^−9^–1.25 × 10^−7^ m^2^, assuming spherical grains), are predicted to be completely transformed to magnesite in less than a year. In practice, larger porous grains with a higher surface area to volume ratio than spheres would also be transformed in a year.

Existing data suggest that under conditions of near 100% relative humidity, conversion of MgO to Mg(OH)_2_ can occur on the order of hours, indicating that over the course of a year the hydration reaction is not rate limiting^43^. This conversion is further dependent on the specific surface area and relative humidity of the system (or water vapor partial pressure) with higher partial pressures resulting in faster conversion. Since conversion of MgO to Mg(OH)_2_ in the presence of water is much faster than the rate of carbonation of Mg(OH)_2_, we assume the carbonation step is rate limiting. Thus, the rate of carbonation can be assumed to be the effective rate for the system.

**Fig. 1:**
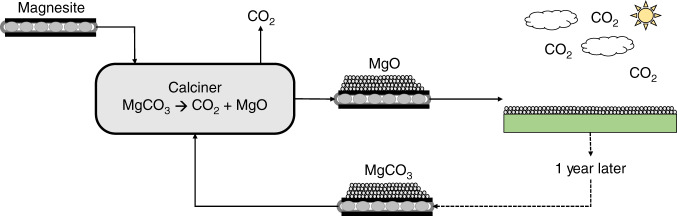
Simplified schematic of the MgO looping process. The initial magnesite feedstock is fed into the calciner where the mineral is heated to produce CO_2_ and MgO. The produced MgO is then transported to land plots where it is deposited and allowed to carbonate over a year. The weathered material is then recollected, primarily in the form of magnesium carbonate, and transported back to the calciner. Here, the material is fed to the calciner with additional magnesite feedstock to make up for environmental losses from the previous cycle. In the calciner, the material is once again heated to produce CO_2_ and MgO. The process is then repeated.

Based on these considerations, we made the conservative assumption that 20 µm particles of caustic magnesia achieve 90% carbonation in a year. The number of carbonation plots in our analysis is optimized to keep the calciner continuously operational, avoiding startup and shutdown expenses. Overall, the process analyzed here is divided into five main steps: mineral acquisition, magnesite calcination, onsite transportation, caustic magnesia carbonation, and mineral recollection (Fig. 1). A complete mass and energy balance for the system is shown in Supplementary Fig. 1 and described in Supplementary Note 1.

The major process assumptions and parameters for the upper and lower bounds of the analysis are outlined in Table 1. Postprocessing of CO_2_ (i.e., compression, transportation, geological sequestration or utilization) is not accounted for in this analysis. The upper and lower bounds correspond to the effect of each parameter on overall process cost, not necessarily the magnitude of each parameter value.

**Table 1:** Assumptions and parameters used for the upper and lower bounds in the process model.

Parameter/Assumption	Value		Comments
	Lower bound	Upper bound	
Calcination			
Calcination temperature [°C]	600	1200	Literature values from 500 to 1200 °C^45^
Calcination time [h]	2	0.5	Literature values from 0.5 to 4 h^45^
Time between calcination loads [h]	0.25	0.25	
Heat of decarbonation [kJ mol^−1^]	118	118	Literature value^70^
Kiln efficiency [%]	90	90	Assumed industry state-of-the-art
Calcination efficiency [%]	90	90	At 600 °C, the decomposition is completed within 2 h. At 800 °C, the decomposition is completed within 30 min^45^. Additional studies show decomposition efficiencies near 90% using 600 and 650 °C for 1.5 h^71^.
Carbonation			
CO_2_ uptake capacity [mol CO_2_ molMgO^−1^]	1	1	Assumed stoichiometric value consistent with magnesite formation
MgO layer thickness [m]	0.1	0.1	
Particle size [µm]	20	20	
Environmental losses [% cycle^−1^]	5	10	
Carbonation efficiency [%]	90	90	
Stirring equipment [acres unit equipment^−1^]	125	125	Literature value^72^
Number of plots	3,504	10,512	Determined to keep the calciner operating continuously at the given calcination conditions
Energy costs and emissions			
Natural gas [$ GJ^−1^]	3.5	3.5	Literature value^7,20^
Natural gas [kgCO_2_ GJ^−1^]	59	59	Literature value^26^
Gasoline [$ gallon^−1^]	2.60	2.60	Average market price for 2019 from EIA^73^
Gasoline [kgCO_2_ gallon^−1^]	8.89	8.89	Literature value from EIA^74^
Grid electricity [$ GJ^−1^]	16.7	16.7	Literature value^7,20^
Grid electricity [kgCO_2_ GJ^−1^]	150	150	Literature value^26^
Solar electricity [$ GJ^−1^]	16.7	16.7	US national average for utility-scale solar ($0.06 kWh^−1^)^44^
Future solar electricity [$ GJ^−1^]	8	8	Projected value ($0.03 kWh^−1^)^44^
Solar electricity [kgCO_2_ GJ^−1^]	6.9	6.9	Literature value^7^
Raw material (mining) emissions [kgCO_2_ tMineral^−1^]	10	10	Literature values from 2 to 12.1^26^
Economic parameters			
Capacity factor [%]	90	90	Consistent with Keith et al.^20^
Plant economic lifetime [yr]	20	20	Consistent with Keith et al.^20^
Discount rate [%]	4	11	
Capital recovery factor [%]	7.4	12.6	Similar to 7.5 and 12.5% used in Keith et al.^20^

Three scenarios are explored in this analysis, related to the type, cost and emissions of electricity used. The first scenario uses grid electricity, assuming electricity is taken directly from the commercial grid. The second scenario uses solar electricity, assuming electricity is obtained via utility solar plants at the current market price. The third scenario uses a projected cost of solar electricity, assuming a decrease in utility solar electricity cost by 2030 as projected by the DOE^44^. The cost of electricity and associated emissions for each scenario are outlined in Table 1.

For this analysis, magnesite is considered to be the feedstock material, with 50,000 tons of raw mineral per carbonation plot. The emissions from mining magnesite are 10 kgCO_2_ tMgCO_3_^−1^, within the high end of the typical range of 1.3–12.5 kgCO_2_ tmineral^−1^^26^. The process costs are not sensitive to the feedstock cost or to the mining emissions, due to repeated reuse of MgO from the feedstock. For this analysis, it is assumed the feedstock is available at the desired particle size of 20 µm or that this particle size is attained in the first calcination step.

Magnesite feedstock is generally calcined at 500–1200 °C^45^. For this analysis, two sets of calcination conditions are used: 600 °C for 2 h (lower bound) and 1200 °C for 0.5 h (upper bound). Calcining at 600 °C for 2 h yields a higher specific surface area (93.07 m^2^ g^−1^ for a 2–5 mm feed precalcination), which aids in subsequent carbonation reactions^45^. Calcination at 1200 °C for 0.5 h results in a decreased surface area (10.9 m^2^ g^−1^ for a 2–5 mm feed precalcination). Calcination yields MgO and a high-purity stream of CO_2_. The calciner is continuously operational with a capacity factor of 90% to account for routine maintenance. The number of carbonation plots was determined to meet this operational capacity. Additionally, an oxy-fired calciner was used. The oxy-fired calciner requires two additional pieces of equipment: an air separation unit (to feed high-purity oxygen to the system) and a condenser (to condense water from the calciner exhaust stream).

Combustion energy and CO_2_ outputs are estimated for oxidation of pure methane. Following combined combustion and calcination, the gas stream is fed into the condenser where water is removed. Since oxy-fired calcination is used, the flue gas is mainly composed of CO_2_ and water vapor, yielding a high-purity stream of CO_2_ after H_2_O condensation. The condensation step produces 0.3 tonnes of H_2_O per tCO_2_ captured from air, which can be sold as a byproduct. CO_2_ removed from Mg-carbonates, and CO_2_ produced from combustion, can be compressed and permanently stored or sold. The cost of compression—not included in our analysis—may add ~$8 tCO_2_^−1^ to the net removed cost, depending on proximity to, and infrastructure at, a storage site^7^.

To move calcined MgO to the plot of land used for weathering, an electric conveyor is used, as is ubiquitous in the mineral extraction industry. These conveyors act as connections between the calcination plant and carbonation plots for spreading MgO. Additionally, the conveyors will transport weathered, carbonated product from the plots to the calcination plant each year. A potential layout of the carbonation plots is shown in Supplementary Fig. 2. Transportation operating costs are related to electricity used by the conveyor system which was determined using motor power details for commercially available mining conveyors (373 kW (500 HP) with a capacity of 454 t h^−1^)^46^.

Weathering in this process takes place on land at ambient conditions. MgO is spread on land in layers 0.1 m thick and stirred daily. Since stirring equipment for large plots of land is not industrially available, values for the capital costs of this equipment are approximated using costs of large-scale agricultural tillage equipment (Table 2).

**Table 2:** Estimated capital expenditures (CAPEX) for the MgO looping process.

CAPEX	Cost [M$]		Comments
	Lower bound	Upper bound	
Raw mineral	8,760	110,376	*Lower bound*: Estimated based on open pit mining cost ($10 tonne^−1^)^75^ and inflated to represent the required size reductions. *Upper bound:* cost of calcined magnesia^76^.
Air separation unit and condenser^a^	785	2,260	*Lower bound*: scaled from Keith et al. installed calciner cost^20^. *Upper bound:* scaled from NASEM Report (built-in installation costs)^7^.
Oxy-fired calciner^a^	930	12,202	*Lower bound:* Scaled from Keith et al.^20^. *Upper bound:* Scaled from NASEM Report (Built-in 4.5× factor for new technology)^7^.
Land	129	1,796	*Lower bound:* Pasteur farm real estate at $1390 per acre. *Upper bound:* Farm real estate at $6430 per acre^54^.
Transportation (Conveyor System)^a^	129	1050	Price from mining cost data^46,77^. *Lower bound*: 1.5× factor to account for commercial possibility. *Upper bound*: 4.5× factor to account for new technology application.
Stirring equipment	28	84	Price quote for industrial farming equipment with a 1.5× factor for new equipment application.
Recollection equipment	22	67	Price quote for industrial farming equipment with a 1.5× factor for new equipment application.
Total CAPEX [M$]	$10,783	$127,835	
CAPEX Annualized [M$ year^−1^]	$794	$16,053	CRF of 7.4% used for lower bound and 12.5% used for upper bound.
CO_2_ capture from air [GtCO_2_ year^−1^]	0.064	0.18	Only includes CO_2_ captured directly from the air.
Total CO_2_ capture [GtCO_2_ year^−1^]	0.12	0.34	Includes CO_2_ captured directly from the air and produced via calcination.
CAPEX [$ tCO_2_^−1^ captured]	$12	$89	
CAPEX [$ tCO_2_^−1^ produced]	$7	$47	

^a^Assumed a scale-up factor^68^.

The system analyzed here has between 3504 (lower bound) and 10,512 (upper bound) carbonation plots, each with ~21,500 tMgO from the original 50,000 tMgCO_3_ feedstock. The number of carbonation plots is optimized for continuous calciner operation. Since the upper bound and lower bound have 30-min and 2-h calcination cycles, respectively, more plots are processed per year in the upper bound scenario. Since each plot is populated with MgO at different times during the year, they will also be recollected and calcined at different times. Additionally, it was assumed that 90–95% of the MgO will be recollected as MgCO_3_ or unreacted MgO, while 5–10% of this material will be lost to the environment. We calculate that losses of MgCO_3_ will be 0.03–0.05% per year and MgO losses will be 3–4% per year (assumptions and results in Supplementary Note 2).

For recollection and delivery to conveyors, the associated pieces of equipment are assumed to be commercially available front-loading tractors. The conveyors will bring MgCO_3_ from the carbonation plots back to the calcination plant and the MgO will be regenerated in the calcination reactor for continued use. By staggering plot maturation times, the central calcination equipment can be used continuously for multiple carbonation plots throughout the year. This also allows for more CO_2_ capture without increasing the required operational scale (i.e., equipment sizing or throughput).

After undergoing repeated calcination, sintering may have a significant effect on MgO reactivity^47^. Studies evaluating capacity losses for magnesium-based adsorbents suggest that after ten cycles, CO_2_ uptake capacity diminishes by 5–7%^48,49^. This would correspond to a capacity loss between 2 and 17% over the plant lifetime, depending on the amount of makeup material and the number of cycles undergone by material lost to the environment (assumptions and results in Supplementary Table 1 and Supplementary Note 3). This analysis assumes 5–10% losses in each cycle, accounting for both environmental losses and possible sintering effects so the initial MgO lasts for 10–20 cycles. The periodic replacement of MgO via addition of MgCO_3_ feedstock is included in the system operating costs as makeup minerals, but capacity losses are not accounted for.

### Cost calculations

The cost estimates presented in this section include capture of CO_2_ from ambient air and subsequent evolution of CO_2_ via a mineral calcination process, not including postprocessing CO_2_ costs. The largest contributions to the capital costs of the system are raw material costs at 81–86% of capital costs, the oxy-fired calciner at ~10% of capital costs, and the air separation unit and condenser at 2–7% of the capital costs. The costing method and scaling factor used for each piece of equipment are presented in Table 2.

Each capital cost value is scaled to the individual process conditions. Here, the upper bound is processing 0.18 Gt CO_2_ year^−1^ using 10,512 carbonation plots, while the lower bound is processing 0.06 Gt CO_2_ year^−1^ using 3504 carbonation plots. Since the upper bound is processing about three times more CO_2_ than the lower bound, the capital cost per tonne CO_2_ is significantly less for the lower bound compared to the upper bound.

Table 3 shows the energy requirements and energy type for each unit operation. The main energy demand of the process is for calcination, which depends on calcining temperature. Therefore, the energy requirements per tonne CO_2_ vary between the lower and upper bounds.

**Table 3:** Energy requirements for the MgO looping process.

Unit operation	Energy requirements		Energy type
	Lower bound	Upper bound	
Air separation unit and condenser^7^ [MJ tCO_2_^−1^]	300	300	Electricity
Oxy-fired calciner [MJ tCO_2_^−1^]	5890	7970	Natural gas
Transportation [MJ tCO_2_^−1^]	8	9	Electricity
Stirring equipment [gallons tCO_2_^−1^]	0.29	0.31	Gasoline
Recollection equipment [gallons tCO_2_^−1^]	0.0051	0.0054	Gasoline

**Fig. 2:**
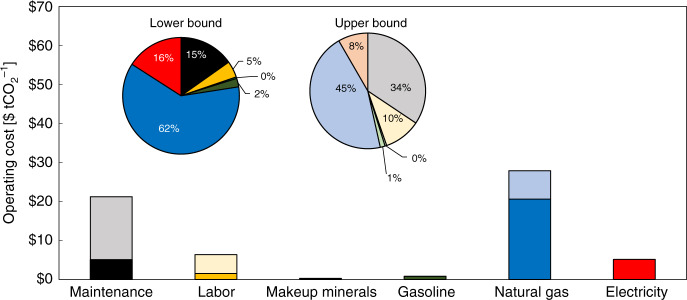
Distribution of operating costs for the enhanced weathering system. Color gradient indicates the difference between the lower and upper operating cost values. Two pie charts are presented representing the operating cost distribution for both the lower (darker color palette) and upper (lighter color palette) bounds of the analysis. Source data are provided as a Source Data file.

Table 4 details the operating costs for the MgO looping system, while Fig. 2 illustrates the breakdown of operating costs by type. There are no variations in the cost between grid and solar electricity scenarios as the cost of electricity is identical. Variations between these energy resource scenarios arise when considering CO_2_ emissions.

**Table 4:** Operating expenditures (OPEX) for the MgO looping process.

OPEX	Cost [M$]		Comments
	Lower bound	Upper bound	
Maintenance	323	3,835	Calculated at 0.03 of total capital^7^
Labor	97	1,151	Calculated at 0.3 of maintenance^7^
Makeup minerals	9	53	
Gasoline	50	151	
Natural gas	1,310	5,036	
Electricity	327	929	Using a value of $16.70 GJ^−1^ for both solar^44^ and grid^20^ electricity
Total OPEX [M$]	$2,117	$11,154	
Total OPEX [$ tCO_2_^−1^ captured]	$33	$62	
Total OPEX [$ tCO_2_^−1^ produced]	$17	$32

The largest contribution to operating costs is the natural gas required to power the calciner, making up 45–62% of operating costs for all scenarios. This indicates process operating costs are sensitive to the price of natural gas. Additionally, electricity makes up 8–16% of operating costs. Other major contributions to operation costs are maintenance (15–34%) and labor (5–10%), which are directly correlated to capital costs.

### Cost of CO_2_

The cost of CO_2_ combines the capital and operating costs presented in the previous sections to develop a process cost per tCO_2_ as outlined in the “Methods” section. These costs are shown in Table 5. In addition, costs for a smaller scale system (10,000 tMgCO_3_ per plot) and reduced layer thickness (0.01 m) are provided in Supplementary Tables 2 and 3, respectively. The sensitivity of process costs to key parameters is shown in Supplementary Fig. 3 and discussed in Supplementary Note 4.

**Table 5:** Summary of CO_2_ capture costs for the MgO looping process using costs as defined in the section “Calculating the cost of CO_2_”.

	Grid electricity	Solar electricity $0.06 kWh^−1^	Solar electricity $0.03 kWh^−1^
Capture cost [$ tCO_2_^−1^]	46–151	46–151	43–148
Net removal cost [$ tCO_2_^−1^]	48–159	46–152	43–149
Produced cost [$ tCO_2_^−1^]	24–79	24–79	23–77

While the cost of capture for the solar electricity scenario is the same as for the grid electricity scenario, the cost of CO_2_ net removed is ~4% less for the solar scenario compared to the grid scenario. This minor difference is due to the lower CO_2_ emissions associated with solar versus grid electricity. Additionally, when accounting for projected cost reduction of solar electricity, the CO_2_ net removed process cost is reduced by ~7% compared to grid electricity.

## Discussion

This study explores a process by which a magnesite feedstock can be repeatedly calcined and carbonated to remove CO_2_ from air, to evaluate the feasibility of this process as a DAC technology. Here, we discuss important factors affecting feasibility, including estimated costs, opportunities for cost reduction, land use, magnesite availability and potential use of alternative feedstocks.

For the process presented here, the cost of CO_2_ net removed ranges from $46 to $159 tCO_2_^−1^ using current costs of grid and solar electricity, while the cost of CO_2_ produced ranges from $24 to $79 tCO_2_^−1^. Using future cost projections for solar electricity yields $43–$149 tCO_2_^−1^ net removed and $23–$77 tCO_2_^−1^ produced. These estimates can be compared to published values for other processes. Currently, DAC processes using synthetic sorbents or solvents have been demonstrated on pilot scales, with costs of CO_2_ net removed reported to be $500–$600 tCO_2_^−1^ captured using low-carbon energy^50^. Aside from industrial-scale initiatives, estimated costs of DAC technologies using combined carbonation and calcination processes have been described. The American Physical Society (APS) estimated a cost of $610–$780 tCO_2_^−1^ net removed for an aqueous calcium looping system using sodium hydroxide and a cost of electricity of $71 MWh^−1^ (or $19.7 GJ^−1^)^2^. By varying packing materials and optimizing the process around this new material, Mazzotti et al.^51^ estimated $510–$568 tCO_2_^−1^ net removed for a similar process. Using natural gas thermal and electric energy, the National Academies of Science, Engineering and Medicine (NASEM) estimated the cost of solvent- and sorbent-based DAC systems tCO_2_^−1^ net removed as $199–$357 tCO_2_^−1^ and $124–$407, respectively^7^. When using solar electric energy and natural gas thermal energy, these values become $165–$295 and $113–$326 tCO_2_^−1^ net removed, respectively. For a process using potassium hydroxide and calcium oxide, Keith et al. estimated costs ranging from $94 to $232 tCO_2_^−1^ captured with a cost of electricity of $30 MWh^−1^ ($8.34 GJ^−1^) and $60 MWh^−1^ ($16.7 GJ^−1^). The $94 tCO_2_^−1^ captured cost is for an *N*th-of-a-kind plant optimized specifically for air-to-fuels^20^, and is approximately the same as the estimated cost of removing CO_2_ from air via MgO looping in this study.

In summary, CO_2_ removal from air via the MgO looping process described in this paper could have a similar or lower estimated cost compared to published estimates for CO_2_ removal using DAC with synthetic sorbents or solvents, within the uncertainties for all of these techniques. Additionally, the proposed process integrates CO_2_ capture from oxy-fired calcination. This reduces the produced cost of CO_2_ and provides a competitive price, if produced CO_2_ were to be sold for use.

As technology continues to develop, there are multiple opportunities to reduce the cost of the enhanced weathering process analyzed here, perhaps most notably by using a solar calciner. The calcination step requires the most process energy. As an alternative to capturing emissions associated with oxy-fired calcination, solar calcination may largely avoid these emissions. Currently, solar calcination is not an industrially available technology. However, research is currently underway to develop high temperature solar kilns for calcination^52,53^. Additionally, adoption and scale-up of industrial-scale electric calciners may allow for low-carbon energy to power the calcination step. Incorporating an experimental calcination process would increase initial capital investment. However, when solar or electric calcination technology becomes established, it could provide a lower-cost, sustainable alternative to oxy-fired calcination, aiding the transition away from fossil fuels.

The process evaluated in this study uses carbonation plots, each with ~21,500 tMgO in a layer 0.1 m thick, using 11 ha of land. For comparison, a small family farm in the US has an average size of 93 ha while large family farms average ~600 ha^54^. Using this approximation, 0.15–0.9 MtCO_2_ year^−1^ could be removed from air on a family farm, equivalent to 160 kgCO_2_ m^−2^ yr^−1^. For additional comparison, biomass-based production usually removes between 1 and 10 kgCO_2_ m^−2^ yr^−1 ^^55^. The upper bound in this analysis would require 0.11 Mha (sequestering 180 million tCO_2_), while the lower bound would require 0.04 Mha (sequestering 64 million tCO_2_). To sequester 1 GtCO_2_ would require ~0.61 Mha (6100 km^2^) of land area.

The land area for electricity generation can also be incorporated into this analysis. The process consumes 0.3 GJ of electricity per tCO_2_ captured. The land area footprint for electrical generation, using natural gas combined cycle (NGCC) methods, is 0.14 ha MW^−1^, while for solar electricity the land requirement is 12.7 ha MW^−1 ^^56^. Therefore, the upper bound would require 290 ha for NGCC electricity and 26,100 ha for solar electricity to remove 180 million tCO_2_ from air. The lower bound would require 91 ha for NGCC electricity and 8300 ha for solar electricity to remove 64 million tCO_2_. To remove 1 GtCO_2_ from the air per year with NGCC would require ~1500 ha (15 km^2^) for electrical generation, while a solar electricity generation would require ~0.14 Mha (1400 km^2^). The land energy requirements for the system coupled to solar and grid electricity are shown in Table 6.

**Table 6:** Land area requirements for the MgO looping process.

	Lower bound	Upper bound	1 GtCO_2_
CO_2_ captured [MtCO_2_ yr^−1^]	60	180	1000
Total plot land area [Mha]	0.04	0.11	0.61
Grid electricity land area [ha]	91	286	1,500
Solar electricity land area [ha]	8,300	26,100	138,000
Total land area (grid) [Mha]	0.04	0.11	0.61
Total land area (solar) [Mha]	0.05	0.14	0.75

In total, the lower and upper bound facilities using natural gas as the source for thermal and electrical requirements have a land area footprint between 0.04 and 0.11 Mha respectively. A facility using solar electricity and natural gas thermal energy has a land area footprint between 0.05 and 0.12 Mha for the lower and upper bounds. The small difference between the land area footprints arises from the small contribution of the footprint for electrical generation to the overall land area requirement (~2% for NGCC electricity and ~18% for solar electricity). To remove 1 GtCO_2_ from air requires 0.61 Mha (~6100 km^2^) for natural gas electricity and thermal energy and 0.75 Mha for solar electricity and natural gas thermal energy (0.02% of the 3.25 billion ha of global marginal land^57^).

These values are similar to the estimated footprints of DAC with synthetic sorbents or solvents^7^. To put these requirements into context, the Nevada Test Site plus the surrounding Nevada Test & Training Range occupy 15,000 km^2^, roughly enough to remove 2.5 GtCO_2_ from air per year via weathering of MgO. That area is about 5.2% of Nevada (286,380 km^2^), 0.15% of the USA (9,833,520 km^2^)^58^, 0.05% of global marginal land (~32,000,000 km^2^)^57^ or 0.01% of global land area (127,343,220 km^2^)^59^.

While this process would never be done on arable land, we used the cost of arable land here because price estimates are readily available. Additionally, our comparison to farm size is for illustrative purposes only. Since arable land is in high demand and is essential for food supplies, our proposed process would use inexpensive marginal land, so that our approximation yields an upper bound cost. Moreover, our upper bound for the cost of land in this analysis is still <1% of the overall capital costs.

When estimating magnesite requirements for this system, there are two main considerations: the initial supply of magnesite to each carbonation facility and the makeup supply of magnesite each subsequent year of facility operation. For the initial supply of magnesite, there are two cases: the lower bound utilizing 3,504 carbonation plots with 5% environmental losses and the upper bound utilizing 10,512 carbonation plots with 10% environmental losses. For both cases, the initial plots are each populated with 50,000 tMgCO_3_. The upper bound requires 525 MtMgCO_3_ to capture 180 MtCO_2_, or 6.2% of global reserves (estimated to be 8.5 billion tons of known, economically and legally producible magnesite)^60^. A graphical representation of global magnesite reserves by country is provided in Supplementary Fig. 4. Additionally, with 10% environmental losses, the lower bound process would require 53 MtMgCO_3_ in replacement magnesite each year or 0.6% of global reserves.

For the lower bound, the initial mineral requirement is 175 MtMgCO_3_ or 2% of global magnesite reserves to capture 64 MtCO_2_. For makeup minerals, the lower bound assumed 5% environmental losses, corresponding to an additional 8.7 MtMgCO_3_ per year or 0.1% of global reserves. Removing 1 GtCO_2_ from air per year would initially require 2.9 GtMgCO_3_ or roughly 29% of global magnesite reserves. The makeup supply would require between 0.15 and 0.29 GtMgCO_3_ per year, or roughly 1.7–3.4% of global magnesite reserves.

Magnesite is not the only mineral that can be used in this process. Another potential source of alkalinity is sodium carbonate, Na_2_CO_3_. According to the US Geological Survey, global reserves are about 25 billion tons, of which about 60 wt% is Na_2_O ^60^. If all of this were used for CO_2_ removal from air, that would yield almost 10 GtCO_2_ per year. It is not clear how practical this might be, since sodium carbonate is very soluble in water, and only preserved in arid climates. Additionally, alkaline industrial wastes may be able to remove ~1 GtCO_2_ per year, based on current production, and this may increase to more than 3 GtCO_2_ capacity per year by 2100^14^.

Limestone (CaCO_3_) and dolomite (CaMg(CO_3_)_2_) are highly abundant, much less labile sources of alkalinity. The reserves of rock commodities are such that they are simply described as very large by the US Geological Survey. Hayes and Waldbauer^61^, estimate that the inventory of sedimentary carbon in the Earth’s crust is ~10^22^ moles. Much more than 10% of this is in limestone and dolomite, corresponding to more than 10^8^ billion tons of rock, about half of which is CaO and MgO.

Finally, or in parallel, Mg-silicates from ultramafic rocks—olivine-rich peridotite from the Earth’s mantle and its hydrated equivalent, serpentinite—could be used as a source of MgO. For simplicity, peridotite can be simplified as nearly pure Mg-olivine (Mg_2_SiO_4_), and serpentinite as serpentine (Mg_3_Si_2_O_5_(OH)_4_) and brucite (Mg(OH)_2_). There are abundant legacy tailings of partially hydrated (serpentinized) peridotite, and the on-land resource of peridotite and serpentinite exceeds 100–1000 trillion tons within 3 km of the surface^10^. There is a large literature on heat-treating serpentinite to create a reactive material for CO_2_ capture and storage^62–67^. To produce MgO for weathering as envisioned in this paper, one would calcine serpentinite, driving off H_2_O and minor CO_2_, to create reactive material composed of MgO and amorphous Mg_3_Si_2_O_7_. After a few weathering and calcining cycles, this would become MgO and SiO_2_. One could then use MgO as described throughout this paper.

In summary, there are many natural sources of alkalinity (MgO, CaO, Na_2_O) that could be weathered and calcined, to remove CO_2_ from air as described in this paper. For example, the US Geological Survey reports that resources from which magnesium compounds can be recovered range from large to virtually unlimited and are globally widespread^60^. However, because the various feedstocks listed here undergo calcining and/or weathering at different rates and conditions compared to those for magnesite, additional calculations would need to be performed to investigate the economic feasibility using the alternatives.

CO_2_ removal from air via the process described in this paper has a similar or lower cost than CO_2_ removal using DAC with synthetic sorbents or solvents, within uncertainty of estimates for both techniques. The net removed cost associated with grid electricity ($48–$159 tCO_2_^−1^) is slightly higher than that of solar electricity ($46–$152 tCO_2_^−1^, or $43–$149 tCO_2_^−1^ when incorporating predicted cost decreases) due to the CO_2_ emissions associated with grid electricity. The process is relatively simple and robust and is feasible at a reasonable cost using existing technology. Additionally, the proposed process integrates CO_2_ capture from the oxy-fired calcination unit, so the cost of produced CO_2_, both removed from air and captured from combustion, is competitive with other sources. While addressing the greenhouse gas problem requires permanent storage of huge amounts of CO_2_, this is not currently profitable. For storage, the integrated CO_2_ capture from the calciner will increase the costs associated with storage—with storage costs of approximately $9–$20 tCO_2_^−1^ stored^7^. However, in the short term, sale of CO_2_ may provide an income stream that attracts the investment required to support research and development of a range of technologies for CO_2_ removal from air.

Finally, we wish to emphasize that DAC technologies, including the MgO looping process analyzed in this paper, are not as effective as point source emissions reductions. However, CO_2_ removal from air will probably be required to limit global warming to less than 2 °C by 2100. In this context, MgO looping offers a practical and relatively inexpensive carbon dioxide removal method.

## Methods

### Equipment scaling and capital costs

Major unit operations in this process include: physical preprocessing, air separation, condenser, oxy-fired calciner, land use, and transportation equipment. Costs for these were developed from two sources. First, quotes from industry for available equipment were used. This includes pricing for raw materials, the transportation conveyor system, and stirring and recollection equipment. For novel or new types of equipment, capital costs were determined based on corresponding literature values. Both of these types of equipment costs were scaled to process 50,000 tMgCO_3_ feedstock per cycle per plot (or 18 ktCO_2_ cycle^−1^ plot^−1^) using the relationship in Eq. (3).

3 $$\frac{{{\mathrm{Cost}}_1}}{{{\mathrm{Cost}}_2}} = \left( {\frac{{{\mathrm{Scale}}_1}}{{{\mathrm{Scale}}_2}}} \right)^\alpha,$$

where *α* is a scaling factor. Traditionally, 0.60 is used as a scaling factor for most industrial equipment. However, for reaction vessels, this scaling factor is conventionally 0.68 ^68^. Additionally, to account for the installed cost of the equipment, a multiplication factor was included. This factor was 1.5× for commercially available equipment and 4.5× for new technology or new applications. Finally, capital expenditures for the process were annualized using capital recovery factors of 7.4% for the lower bound and 12.6% for the upper bound.

### Energy requirements and operating costs

Energy costs associated with the process were calculated for each unit operation based on operating time, equipment capacities, and periodic nature of the process. Operations energy requirements were determined for a single carbonation cycle and scaled to the required number of plots. From the energy analysis on each unit operation, operating costs for the system were developed. The operating costs were determined using $16.7 GJ^−1^ for grid and utility solar electricity. Additionally, a third scenario was developed using a projected solar electricity cost of $8 GJ^−1^. Parameters for energy costs and associated emissions are outlined in Table 1.

### Calculating the cost of CO_2_

The costs of CO_2_ determined in this analysis considers three different scenarios. First, a general case is presented where grid electricity is used. Second, the current industrial cost of electricity generated by solar photovoltaic cells was used. Finally, process costs were anticipated using the projected price of solar electricity, which is expected to reach $8 GJ^−1^ ($0.03 kWh^−1^) by 2030^44^. Solar electricity is not entirely carbon-free as there are emissions during fabrication, mainly associated with manufacturing of solar cells^7^. While these emissions are significantly less than for a comparable amount of grid electricity, they must still be included in the analysis.

Three different costs of CO_2_ are calculated for each scenario: the cost of capturing CO_2_, the cost of CO_2_ net removed from the atmosphere, and the produced cost of CO_2_^69^. The cost of capturing CO_2_ is the direct cost of the process and is calculated in accordance with Eq. (4). This value represents how much it would cost for the process to remove 1 tCO_2_ directly from the atmosphere.

4 $${\mathrm{Cost}}[{\mathrm{\$}}\,{\mathrm{tCO}}_2^{ - 1}] = \frac{{{\mathrm{Process}}\,{\mathrm{cost}}\,[{\mathrm{\$ }}]}}{{{\mathrm{CO}}_2\,{\mathrm{capture}}\,{\mathrm{from}}\,{\mathrm{air}}\,[{\mathrm{tCO}}_2]}}.$$

The cost of CO_2_ net removed takes the cost of capturing CO_2_ and adjusts for emissions produced in the process, and not captured onsite. For each step in the process, some amount of energy in the form of electricity, natural gas, or fuel is required. With each energy source, there are associated CO_2_ emissions. We have assumed that electricity will be generated off site, and that the emissions from generation are not captured. Since the goal of the process is to remove CO_2_ from the atmosphere, it is important to consider emissions from the process itself. The cost of CO_2_ net removed includes the emissions as a result of the process and scales the cost of capturing CO_2_ to develop a cost that represents a net capture of 1 tCO_2_ as follows:

5 $$	{\mathrm{Net}}\,{\mathrm{removed}}\,{\mathrm{cost}}[{\mathrm{\$ }}\,{\mathrm{tCO}}_2^{ - 1}] \\ 	\qquad\qquad = \frac{{{\mathrm{Process}}\,{\mathrm{cost}}\,[{\mathrm{\$ }}]}}{{{\mathrm{CO}}_2\,{\mathrm{capture}}\,{\mathrm{from}}\,{\mathrm{air}}\,[{\mathrm{tCO}}_2] - {\mathrm{CO}}_2{\mathrm{released}}\,{\mathrm{by}}\,{\mathrm{process}}\,[{\mathrm{tCO}}_2]}}.$$

Finally, the produced cost of CO_2_ uses the cost of capturing CO_2_ and adjusts for additional CO_2_ captured onsite, specifically CO_2_ captured from natural gas combustion in the oxy-fired calcination unit. Ultimately, the produced cost of CO_2_ is equivalent to the price at which CO_2_ could be sold for the process to break-even. This is described in Eq. (6).

6 $$	{\mathrm{Produced}}\,{\mathrm{cost}}\,[{\mathrm{\$ }}\,{\mathrm{tCO}}_2^{ - 1}] \\ 	\qquad= \frac{{{\mathrm{Process}}\,{\mathrm{cost}}\,[{\mathrm{\$ }}]}}{{{\mathrm{CO}}_2\,{\mathrm{capture}}\,{\mathrm{from}}\,{\mathrm{air}}\,[{\mathrm{tCO}}_2] + {\mathrm{additional}}\,{\mathrm{CO}}_2\,{\mathrm{captured}}\,{\mathrm{by}}\,{\mathrm{process}}\,[{\mathrm{tCO}}_2]}}.$$

### Sensitivity analysis

A sensitivity analysis was performed on the proposed system with respect to six important parameters: carbonation efficiency, environmental losses, calcination temperature, calcination time, and the number of carbonation plots. The sensitivity analysis was performed in MATLAB by iteratively solving the process simulation between designated parameter values. This was performed for both the lower and upper bounds of the analysis. The complete sensitivity analysis is presented in Supplementary Fig. 3 and described in Supplementary Note 4.

### Reporting summary

Further information on research design is available in the Nature Research Reporting Summary linked to this article.

## Supplementary information

Supplementary Information

Reporting Summary

## Data Availability

The authors declare that the data supporting the findings of this study are available within the paper and its supplementary information files. The source data underlying all figures and tables in both the main text and supplementary information is provided as Supplementary Data.

## Source data

Source Data
