# Supplementary material for: Ambient weathering of magnesium oxide for CO2 removal from air
Source: Nat Commun. 2020 Jul 3;11:3299. doi: 10.1038/s41467-020-16510-3 (PMC7335196; doi:10.1038/s41467-020-16510-3)
Supplement: Supplementary file 1 — Supplementary Information [file 41467_2020_16510_MOESM1_ESM.pdf]

Supplementary Materials for

Ambient Weathering of Magnesium Oxide for CO_2 Removal from Air

McQueen et al.

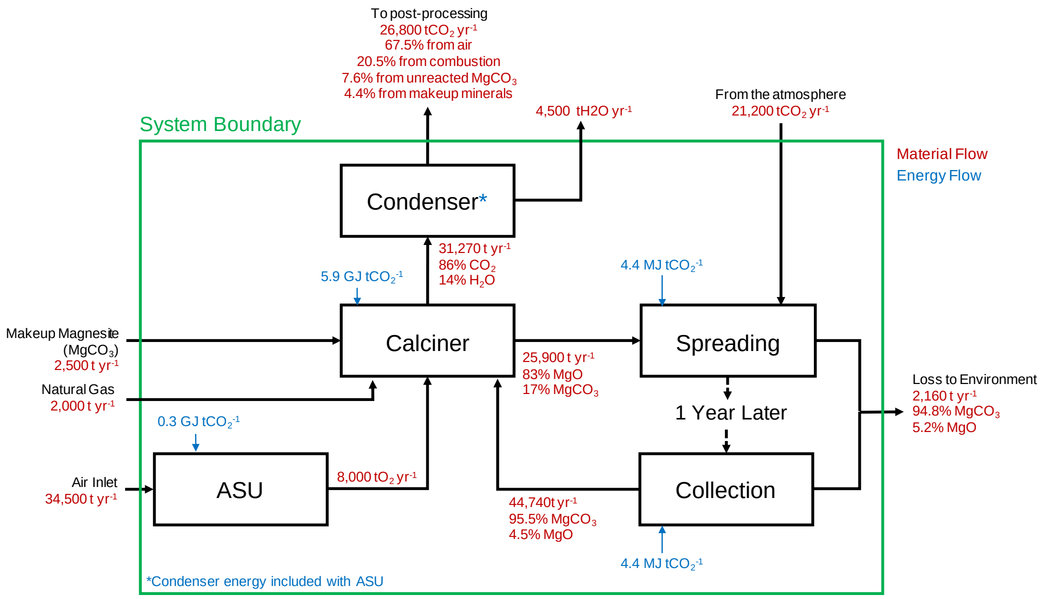

Supplementary Figure 1: Material and energy flow diagram for the proposed MgO looping process. The diagram here is representative of the lower bound scenario operating at steady-state. The mass flows are on a per plot basis.

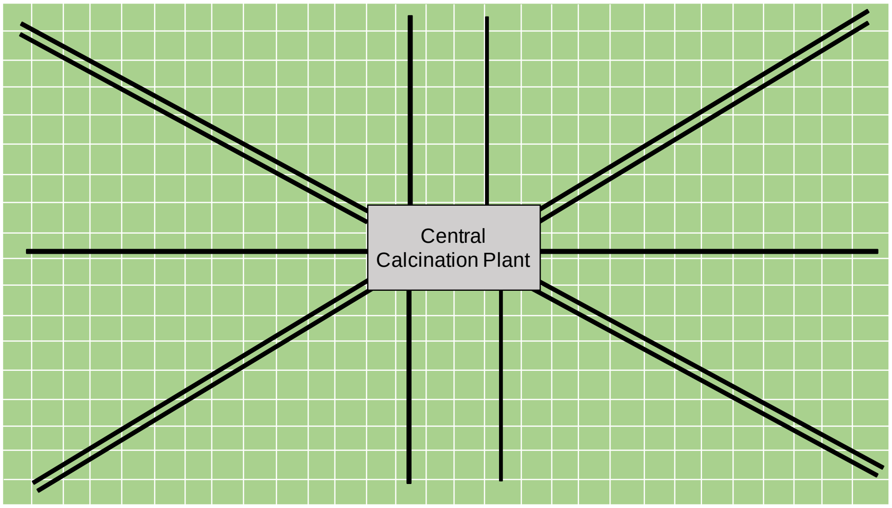

Supplementary Figure 2: Potential layout for the calcination facility with n carbonation plots. Here, n indicates the number of carbonation plots corresponding to the number of plots necessary for continuous operation of the calciner with a 90% capacity factor. The solid black lines represent the conveyor system between the calcination plant and the carbonation plots.

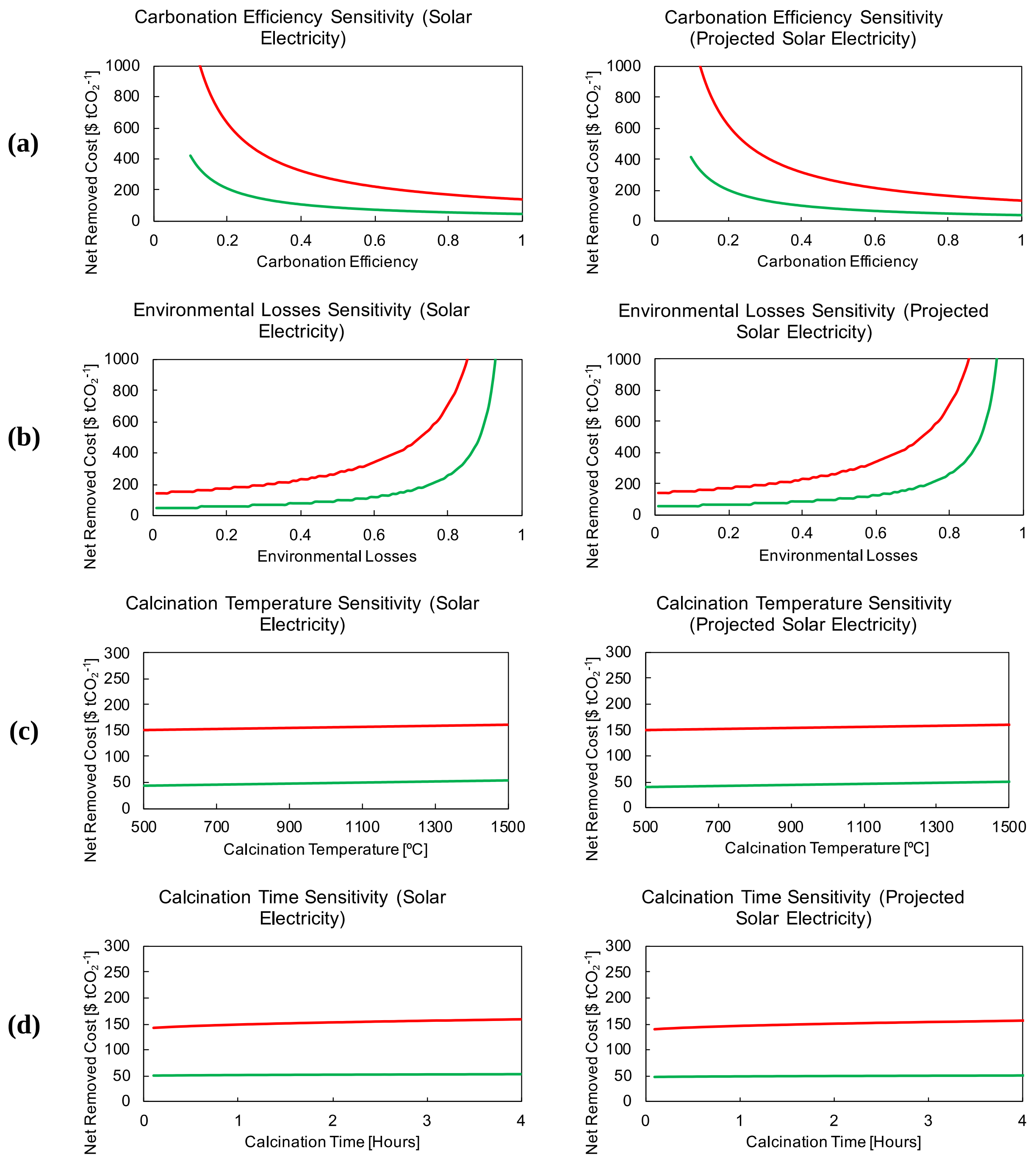
(a)
Carbonation Efficiency Sensitivity (Solar Electricity)
Carbonation Efficiency Sensitivity (Projected Solar Electricity)
Net Removed Cost [$ tCO2-1]
Carbonation Efficiency
(b)
Environmental Losses Sensitivity (Solar Electricity)
Environmental Losses Sensitivity (Projected Solar Electricity)
Environmental Losses
(c)
Calcination Temperature Sensitivity (Solar Electricity)
Calcination Temperature Sensitivity (Projected Solar Electricity)
Calcination Temperature [ºC]
(d)
Calcination Time Sensitivity (Solar Electricity)
Calcination Time Sensitivity (Projected Solar Electricity)
Calcination Time [Hours]

(e)

(f)

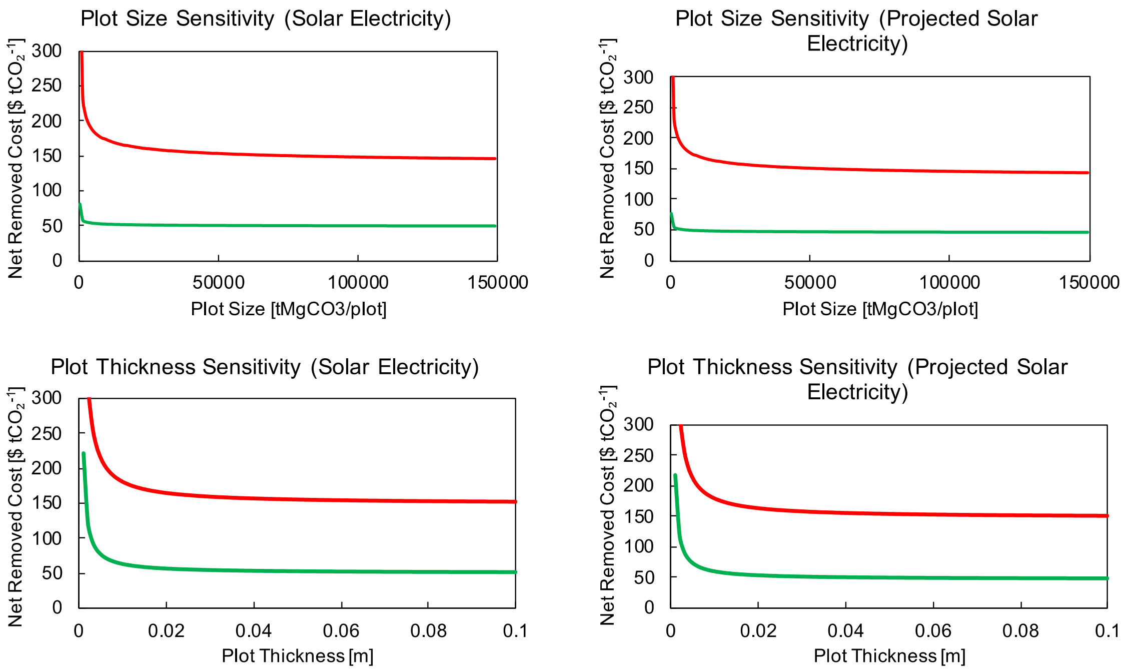

Supplementary Figure 3: Sensitivity analysis for 5 parameters: (a) Carbonation Efficiency, (b) Environmental Losses, (c) Calcination Temperature, (d) Calcination time, (e) Scale (Inlet $MgCO_3$ Flow per Plot), and (f) Plot Thickness. The costs presented here represent the solar electricity scenario (left-hand side) and the projected solar electricity scenario (right-hand side). The green and red lines represent the lower and upper bounds, respectively. Source data are provided as a Source Data file.

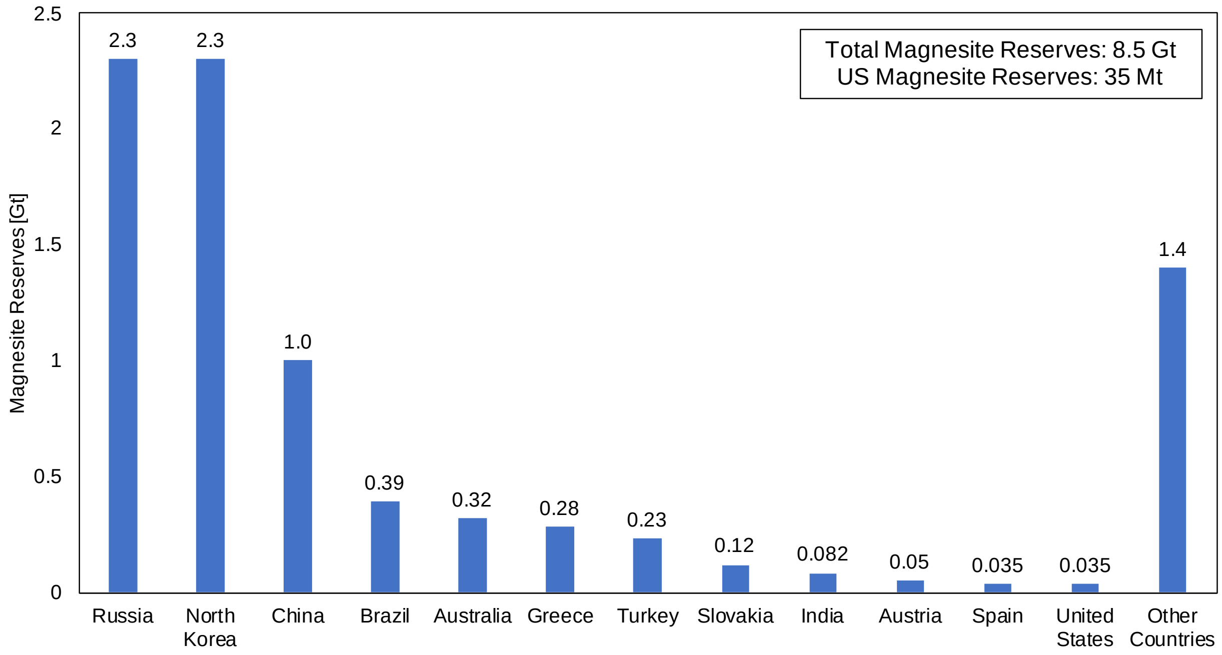

Supplementary Figure 4: Global magnesite reserves by country.[3] Source data are provided as a Source Data file.

Supplementary Table 1: Capacity decrease over the system lifecycle

	Capacity Decrease	**Percent Original CO_2 Uptake Maintained in Last Cycle**	
	[%/cycle]	Lower Bound	Upper Bound
All environmental losses of oldest material	0.50%	90%	98%
	0.70%	89%	97%
All environmental losses of newest material	0.50%	87%	92%
	0.70%	83%	89%

Supplementary Table 2: Summary of CO_2 capture costs for the MgO looping process for an initial plot populated from 10,000 tonnes of $MgCO_3$, using costs as defined in Section 4.3 of the main article

	Grid Electricity	Solar Electricity $0.06 kWh^{-1}	Solar Electricity $0.03 kWh^{-1}
**Capture cost [$ tCO_2^{-1}]**	49 - 163	49 - 163	46 - 160
**Net Removal Cost [$ tCO_2^{-1}]**	51 - 172	49 - 164	46 - 161
**Produced Cost [$ tCO_2^{-1}]**	29 - 85	29 - 85	27 - 84

Supplementary Table 3: Summary of CO_2 capture costs for the MgO looping process for a plot thickness of 0.01 m, using costs as defined in Section 4.3 of the main article

	**Grid Electricity**	**Solar Electricity $0.06 kWh^{-1}**	**Solar Electricity $0.03 kWh^{-1}**
**Capture cost [$ tCO_2^{-1}]**	56 - 174	56 - 174	53 - 171
**Net Removal Cost [$ tCO_2^{-1}]**	60 - 189	58 - 180	55 - 177
**Produced Cost [$ tCO_2^{-1}]**	32 - 91	32 - 91	31 - 90

Supplementary Note 1

Supplementary Figure 1 represents the material and energy flows on a per plot basis. The system will operate with many plots at the same time. However, since the plots are staggered in their maturation time, only one plot will be processed at a time. The number of overall plots was determined to ensure continuous operation of the calciner unit to eliminate costs associated with repeated startup and shutdown.

Further, the lower bound and upper bound analyses differ with respect to the process requirements, specifically related to the temperature for calcination and process throughput. For the purpose of illustrating the mass and energy flows used to develop the process economics, a representative set of conditions are shown in Supplementary Figure 1 for the lower bound. While these calculations and conditions are specifically representing the lower bound scenario, they are easily transferrable for the upper bound scenario by changing the parameters of interest outlined in Table 1 in the main text of this article.

Supplementary Note 2

Soil erosion rates are estimated are estimated as 3 tonnes per hectare per year (with a higher end near 3.88 tonnes per hectare per year).[1] A 10 cm layer of soil with an average bulk density of around 2 t/m^3 will have a mass of around 2,000 tonnes per ha where 3 tonnes is a loss of 0.15%/yr.

The solubility of magnesite and periclase (MgO) was determined using the geochemical code PHREEQC[2] with the Lawrence Livermore National Laboratory database ('thermo.com.V8.R6.230). For magnesite in deionized water at 25ºC, the solubility of Mg is 0.18 mol Mg m^{-3}. For periclase in deionized water at 25ºC, the solubility of Mg is 22.5 mol m^{-3}.

Assuming 500 mm rainfall per year, this is equivalent to 5,000 m^3 ha^{-1} $year^{-1}$. The losses for $MgCO_3$ and MgO are 0.07 $tMgCO_3$ ha^{-1} $year^{-1}$ and 4.5 tMgO ha^{-1} $year^{-1}$. Based on the flowrate per plot of 25,100 tonnes of MgO distributed and 37,000 tonnes of $MgCO_3$ collected, the losses equate to 0.03 – 0.05% of $MgCO_3$ per year and 3 – 4% of MgO per year.

Supplementary Note 3

The literature suggests that there is a 5 – 7% decrease in capacity of the MgO over the first 10 cycles. Assuming that this can be interpolated on a per cycle basis, this results in a capacity loss of 0.5 – 0.7% per cycle.

Two limiting cases were explored. First, the capacity losses were assessed for the upper and lower bound assuming that all of the environmental losses are older MgO with diminished capacity. This would be the upper bound for the analysis – or the ‘best case.’ Second, the capacity losses were assessed for the upper and lower bound assuming that all the environmental losses are the most newly produced MgO. This would be the lower bound for the analysis – of the ‘worst case.’ The results are summarized Supplementary Table 1.

It is likely that the capacity losses for the lower bound would be between 10% and 17% over the 20-year lifetime of the plant and for the upper bound between 2% and 11%. This depends on the amount of makeup MgO introduced into the system to make up for the environmental losses. The capacity losses for the system is most likely in the ranges presented here, but the exact values are unknown.

Supplementary Note 4

A sensitivity analysis was performed on the proposed system with respect to six important parameters: carbonation efficiency, environmental losses, calcination temperature, calcination time, scale (in the form of inlet magnesite feedstock) and plot thickness. The results of this analysis are shown in Supplementary Figure 3 for the solar electricity scenario, as well as the projected cost of solar electricity.

From Supplementary Figure 3(a) it can be seen that cost of CO_2 net removed is highly dependent on the carbon mineralization efficiency (mass fraction of caustic MgO converted to $MgCO_3$ in one weathering step, one year in this analysis). For the scenarios, the net removed cost begins to exponentially increase below 50% efficiency. Since lower carbonation efficiency indicates that less CO_2 is captured by the process, there is less CO_2 to offset the carbon footprint of the process itself. Additionally, it is important to note that the sensitivity to environmental losses exhibits a similar trend to the carbonation efficiency (Supplementary Figure 3(b)). This indicates that the loss of raw material significantly impacts the net removed cost of the system as it requires more frequent replenishment of magnesite feedstock. This increases the operating cost associated with makeup minerals.

Further, the calcination conditions also play a role in the economic viability of the process (Supplementary Figure 3(c) and (d)). Increasing the calcination temperature increases the amount of energy required for the calcination reaction to take place. Therefore, there is a linear trend between the calcination temperature and the net removed process costs, accounting for the increased operating costs. The calcination time changes the scale of CO_2 capture. Since the number of plots are determined to keep the calciner continuously operational (with a 90% capacity factor), the shorter the calcination time, the more plots can utilize the same equipment. Therefore, the system experiences an increase in CO_2 capture capacity with no increase in capital expenses. The calcination time has a larger impact on the overall process cost than the calcination temperature. Therefore, for process optimization purposes, a lower calcination temperature with a short calcination time would be advantageous.

Supplementary Figure 3(e) shows the impact of the operational scale on the overall process cost (in the form of initial magnesite feed in tonnes per plot). The results indicate an exponential decrease in the process cost from scales going from 10 to 10,000 tonnes magnesite per plot. This indicates that the process performs better on a larger scale due to the scaling of capital equipment and ability to capture more CO_2 overall per process facility.

At 10,000 tonnes of magnesite per plot (or 1/5th the size presented in the main article text), the costs increase slightly. These values are shown in Supplementary Table 2.

While these values are close to the values presented in the main body of the report, they result in a reduction in CO_2 captured by the process, as well as the initial capital investment in the process. Populating each of the plots with 10,000 tonne of magnesite results in ~4,300 tonnes of MgO per plot capturing ~3,400 - 3,600 tonnes of CO_2 per plot. This results in the upper bound capturing 36 million tonnes of CO_2 per year and the lower bound capturing 13 million tonnes of CO_2 per year.

This additionally results in a decrease in the gross initial capital investment. For the upper bound the capital investment becomes $28.2 billion, which corresponds to $0.780 billion per million tonnes of CO_2 captured. For the lower bound, the capital investment becomes $2.5 billion, which corresponds to $0.195 billion per million tonnes of CO_2 captured.

The analysis in the main body of the report uses an upper bound of $127 billion to capture 0.18 billion tonnes of CO_2 per year and a lower bound of $10.7 billion to capture 64 million tonnes of CO_2 per year. Resulting in $0.668 billion per million tonnes CO_2 captured for the upper bound and $0.178 billion per million tonnes CO_2 captured for the lower bound.

Finally, Supplementary Figure 3(f) demonstrates the process sensitivity to the plot thickness. It is observed that until thicknesses near 0.01 m (or 1 cm) the price stays fairly consistent. At 1 cm, the costs of the process are shown in Supplementary Table 3. By reducing the plot thickness to 1 cm the costs increase by roughly 15 - 25% for all cases. In the case that diffusion of CO_2 through 10 cm of material is limiting to the process, the layers can be made significantly smaller with only a marginal increase in cost for the overall process.

Supplementary References

1. Borrelli, P. *et al.* An assessment of the global impact of 21st century land use change on soil erosion. *Nat. Commun.* **8**, 1–13 (2017).
2. Parkhurst, D. L. & Appelo, C. *User's guide to PHREEQC (Version 2): A computer program for speciation, batch-reaction, one dimensional transport, and inverse geochemical calculations*. (1999).
3. Statista. Magnesite reserves worldwide by major countries 2018. https://www.statista.com/statistics/264953/global-reserves-of-magnesium-by-major-countries/ (2018).
