# Supplementary material for: Ambient weathering of magnesium oxide for CO2 removal from air
Source: Nat Commun. 2020 Jul 3;11:3299. doi: 10.1038/s41467-020-16510-3 (PMC7335196; doi:10.1038/s41467-020-16510-3)
Supplement: Supplementary file 2 — Reporting Summary [file 41467_2020_16510_MOESM2_ESM.pdf]

nature research

Corresponding author(s): Jennifer Wilcox

Last updated by author(s): Apr 30, 2020

Reporting Summary

Nature Research wishes to improve the reproducibility of the work that we publish. This form provides structure for consistency and transparency in reporting. For further information on Nature Research policies, see Authors & Referees and the Editorial Policy Checklist.

Please do not complete any field with "not applicable" or n/a. Refer to the help text for what text to use if an item is not relevant to your study. For final submission: please carefully check your responses for accuracy; you will not be able to make changes later.

Statistics

For all statistical analyses, confirm that the following items are present in the figure legend, table legend, main text, or Methods section.

n/a	Confirmed	
☒	☐	The exact sample size (n) for each experimental group/condition, given as a discrete number and unit of measurement
☒	☐	A statement on whether measurements were taken from distinct samples or whether the same sample was measured repeatedly
☒	☐	The statistical test(s) used AND whether they are one- or two-sided<br>*Only common tests should be described solely by name; describe more complex techniques in the Methods section.*
☒	☐	A description of all covariates tested
☒	☐	A description of any assumptions or corrections, such as tests of normality and adjustment for multiple comparisons
☒	☐	A full description of the statistical parameters including central tendency (e.g. means) or other basic estimates (e.g. regression coefficient) AND variation (e.g. standard deviation) or associated estimates of uncertainty (e.g. confidence intervals)
☒	☐	For null hypothesis testing, the test statistic (e.g. F, t, r) with confidence intervals, effect sizes, degrees of freedom and P value no<br>*Give P values as exact values whenever suitable.*
☒	☐	For Bayesian analysis, information on the choice of priors and Markov chain Monte Carlo settings
☒	☐	For hierarchical and complex designs, identification of the appropriate level for tests and full reporting of outcomes
☒	☐	Estimates of effect sizes (e.g. Cohen's d, Pearson's r), indicating how they were calculated

Our web collection on statistics for biologists contains articles on many of the points above.

Software and code

Policy information about availability of computer code

Data collection	No software was used for the data collection
Data analysis	MATLAB_R2018B was used to perform calculations associated with the technoeconomic analysis, as well as for running sensitivity analyses.

For manuscripts utilizing custom algorithms or software that are central to the research but not yet described in published literature, software must be made available to editors/reviewers. We strongly encourage code deposition in a community repository (e.g. GitHub). See the Nature Research guidelines for submitting code & software for further information.

Data

Policy information about availability of data

All manuscripts must include a data availability statement. This statement should provide the following information, where applicable:

- Accession codes, unique identifiers, or web links for publicly available datasets
- A list of figures that have associated raw data
- A description of any restrictions on data availability

The authors declare that the data supporting the findings of this study are available within the paper and its supplementary information files. The source data underlying all figures and tables in both the main text and supplementary information is provided as a Source Data file.

Field-specific reporting

Please select the one below that is the best fit for your research. If you are not sure, read the appropriate sections before making your sel[illegible]n.

☐ Life sciences ☐ Behavioural & social sciences ☒ Ecological, evolutionary & environmental sciences

For a reference copy of the document with all sections, see nature.com/documents/nr-reporting-summary-flat.pdf

Ecological, evolutionary & environmental sciences study design

All studies must disclose on these points even when the disclosure is negative.

Study description	This paper analyzes the cost, energy requirements, and CO2 balance for a land-based enhanced weathering cycle utilizing magnesite (MgCO3) feedstock to repeatedly capture CO2 from the atmosphere.
Research sample	This study uses existing data to determine a theoretical cost of CO2 for a novel, land-based enhanced weathering cycle. The data is primarily from two sources: (1) peer reviewed articles available in the literature and (2) reports from authoritative sources (such as the national academies of science, engineering and mathematics (NASEM), or the energy information agency (EIA)). The data sources are referenced directly in the text.
Sampling strategy	There was no sampling procedure used in this analysis.
Data collection	The data collection was performed by reading and reviewing results from peer reviewed articles. Noah McQueen primarily researched process parameters and relationships. All authors contributed sources and data related to their field of expertise.
Timing and spatial scale	The data was not taken within a specified time period.
Data exclusions	No data was intentionally excluded from the analysis.
Reproducibility	The data used to develop the basis for the study is provided within the study text, allowing for the results of the analysis to be reproduced. The analysis is primarily based on information and analyses from existing literature. The sources of assumed values are indicated in the text.
Randomization	Samples/organisms/participants were not allocated into groups in this study. Additionally, there were no physical experiments performed in developing this analysis. Therefore, there was no risk of the order of experiments or the grouping of participants influencing the accuracy of the results presented in this study. The study varies variables between two bounds (upper bound and lower bound) to provide a cost region for which the proposed process may reside in.
Blinding	There were no participants in this study, therefore blinding was not used.

Did the study involve field work? ☐ Yes ☒ No

Reporting for specific materials, systems and methods

We require information from authors about some types of materials, experimental systems and methods used in many studies. Here, indicate whether each material, system or method listed is relevant to your study. If you are not sure if a list item applies to your research, read the appropriate section before selecting a response.

Materials & experimental systems

n/a	Involved in the study	
☒	☐	Antibodies
☒	☐	Eukaryotic cell lines
☒	☐	Palaeontology
☒	☐	Animals and other organisms
☒	☐	Human research participants
☒	☐	Clinical data

Methods

n/a	Involved in the study	
☒	☐	ChIP-seq
☒	☐	Flow cytometry
☒	☐	MRI-based neuroimaging

This checklist template is licensed under a Creative Commons Attribution 4.0 International License, which permits use, sharing, adaptation, distribution and reproduction in any medium or format, as long as you give appropriate credit to the original author(s) and the source, provide a link to the Creative Commons license, and indicate if changes were made. The images or other third party material in this article are included in the article's Creative Commons license, unless indicated otherwise in a credit line to the material. If material is not included in the article's Creative Commons license and your intended use is not permitted by statutory regulation or exceeds the permitted use, you will need to obtain permission directly from the copyright holder. To view a copy of this license, visit http://creativecommons.org/licenses/by/4.0/
